# Effects of gravity and surface tension on steady microbubble propagation in asymmetric bifurcating airways

**DOI:** 10.1063/5.0012796

**Published:** 2020-07-24

**Authors:** Bacha Munir, Yong Xu

**Affiliations:** School of Natural and Applied Sciences, Department of Applied Mathematics, Northwestern Polytechnical University, Shaanxi, Xi’an 710072, People’s Republic of China

## Abstract

Mechanical ventilation is nowadays a well-developed, safe, and necessary strategy for acute respiratory distress syndrome patients to survive. However, the propagation of microbubbles in airway bifurcations during mechanical ventilation makes the existing lung injury more severe. In this paper, finite element and direct interface tracking techniques were utilized to simulate steady microbubble propagation in a two-dimensional asymmetric bifurcating airway filled with a viscous fluid. Inertial effects were neglected, and the numerical solution of Stokes’s equations was used to investigate how gravity and surface tension defined by a Bond (Bo) number and capillary (Ca) number influence the magnitudes of pressure gradients, shear stresses, and shear stress gradients on the bifurcating daughter airway wall. It is found that increasing Bo significantly influenced both the bubble shape and hydrodynamic stresses, where Bo ≥ 0.25 results in a significant increase in bubble elevation and pressure gradient in the upper daughter wall. Although for both Bo and Ca, the magnitude of the pressure gradient is always much larger in the upper daughter airway wall, Ca has a great role in amplifying the magnitude of the pressure gradient. In conclusion, both gravity and surface tension play a key role in the steady microbubble propagation and hydrodynamic stresses in the bifurcating airways.

## I. INTRODUCTION

The recent outbreak of the Covid-19 pandemic has affected a large mass of people throughout the world.^1,2^ It causes acute respiratory distress syndrome (ARDS), which is a dangerous, even fatal, lung disease.^3,4^ In ARDS, the alveolar–capillary membrane ruptures and gets more permeable to the pulmonary fluid. This severely compromises gas exchange between alveoli and bloodstreams and leads to pulmonary edema. Sepsis, hypoxia, severe pneumonia, smoking, and surfactant deficient lungs are some direct and indirect paths to develop ARDS. In this regard, mechanical ventilation (MV) is a modern standard of care for patients suffering from ARDS. Improvements in ventilator-treatment strategies include positive end-expiratory pressure (PEEP) and lower tidal volumes (LTVs); however, the mortality rate from ARDS is still high.^5–8^ During ventilation, oxygen enters the capillaries in the form of microbubbles. The progressive microbubbles then generate hydrodynamic stresses including mechanical forces^9–12^ and exacerbate the lung injury. Ventilator-induced lung injury (VILI) was experimentally and computationally investigated by many researchers. For example, the progression of a semi-infinite bubble^11^ in a collapsed pulmonary airway was studied both experimentally and computationally. Experimental findings showed that the epithelial cells were significantly injured by the progressive semi-infinite bubble, while computational results revealed that the steep and large opposite sign magnitudes of the pressure gradient near the thin bubble tip are responsible for cell damage. With the support of this work, Kay *et al.*^10^ proved that the cell injury does not depend on the semi-infinite bubble exposure duration but is due to the magnitude of the pressure gradient. An *in vitro* experimental model for airway reopening was presented to find the effects of airway diameter and cell confluence on the lung injury.^13^ This study mainly focused on the cell necrosis between the terminal and respiratory bronchioles. They reported that decreasing the reopening velocity and airway radius results in an increase in hydrodynamic stresses and hence cell damage. A three-dimensional (3D) image-based finite element analysis^14^ indicated that the confocal cells can develop less membrane strain than the subconfluent cells during collapsed airway reopening. However, it was found that membrane strain decreases by increasing the cell’s stiffness and cortex region.

The effects of Bond number (Bo), Reynolds number (Re), and capillary number (Ca) on microbubble splitting and cell damage in symmetrically bifurcating airway model were investigated.^15^ It is reported that the pressure gradient is the key component of stresses, responsible for the upper daughter airway epithelial cell injury. In another recent study,^16^ the effects of Bo, Re, and Ca on small bubble splitting through symmetrically bifurcating microvessels were investigated. They identified that vortex-induced shearing is the possible mechanism for endothelial cell damage. Higuita-Castro *et al.*^17^ reported that fiber stiffness and topography highly affect the epithelial/endothelial cell efficiency during fluid occluded airway reopening. The study also showed that the increase in fiber stiffness can alter the cytoskeletal structure, increase tight junction formation, and reduce barrier permeability.

Tavana *et al.*^18^ experimentally and theoretically proved that the surfactants can remarkably decrease the lung injury. Their results indicate that the addition of surfactants can protect the airway wall’s lining cells from damage. Their parallel computational findings also strongly supported the experimental results. The Marangoni effects generated by an infinitely long bubble^19^ with surfactants in a capillary tube were studied. They demonstrated that an increase in the Marangoni effect increased the pressure and wetting-layer thickness. A similar computational study^20^ was conducted for surfactant laden plug progression in neonatal airways. It is observed that, before the addition of surfactants, the maximum magnitudes of pressure and shear stress gradients exist. However, after the addition of the surfactants, the gradients were completely diminished. More recently, Muradoglu *et al.*^21^ observed that even a tiny amount of surfactants can highly reduce the mechanical forces and hence the lung injury. They also concluded that surfactants can delay plug damage and airway reopening.

Earlier studies were only limited to bubble propagation/splitting in straight and symmetrically bifurcating airway models and a corresponding mechanism for epithelial cell damage. In reality, the pulmonary system is very complex and potentially asymmetric, and the cell injury mechanism yet remains unknown. In addition, in the previous studies, the epithelial cell’s damage was strongly correlated with the large and steep opposite sign magnitudes of the pressure gradient^10,15,18^ instead of shear stress gradient magnitudes. Previously, Chen *et al.*^15^ investigated the effects of gravity, inertia, and surface tension on hydrodynamic stresses; however, deep into lungs (i.e., between the terminal and respiratory bronchioles), slow viscous flow exists^13,22^ where the inertial effects may be neglected. A strong motivation for this analysis is based on the experimental work of Yalcin *et al.*^13^ and the complex asymmetric bifurcating structure of the airway tree.^23^ This work aims to numerically elucidate the effects of gravity and surface tension on steady microbubble propagation and hydrodynamic stresses. More specifically, the study mainly focuses on how these factors can influence the pressure gradient at the bifurcating airway walls to explore the possible mechanism for the asymmetric bifurcating airway injury. To carry out successfully this goal, a computational model was developed to simulate microbubble propagation in a two-dimensional (2D) asymmetric bifurcating airway filled with a viscous fluid. Assuming quasi-steady propagation of the microbubble, inertial effects were neglected and Stokes’s equations were solved using finite element techniques (FETs) in COMSOL^24^ for different Bo and Ca values.

## II. PROBLEM STATEMENT

**FIG. 1.**
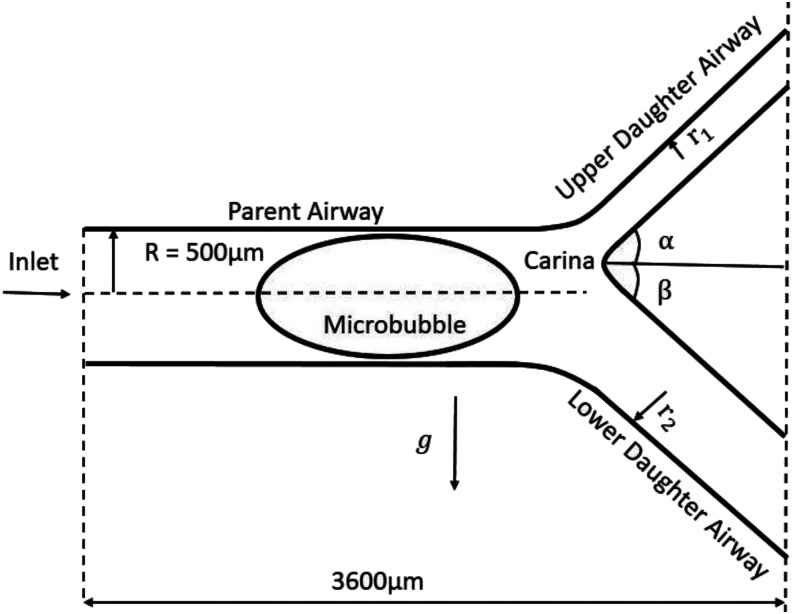
A schematic diagram of the microbubble propagation in the asymmetric bifurcating airway. r_1_ and r_2_ are the radii of the upper and lower daughter branches, respectively.

Consider the steady motion of an initially elliptical shape microbubble in an incompressible Newtonian fluid flowing in an asymmetric bifurcating airway, as shown in Fig. 1. The radius of the parent airway is R = 500 *µ*m, while the radius of the upper daughter airway is half of the lower. The microbubble is initially perfectly elliptical with a semi-major/minor axis of 800 *µ*m and 400 *µ*m, respectively, and its center is located at the origin. The carina is positioned at a point P(1300, 2500), while the branching angle is α = β = 40°. The total length of the airway is 3600 *µ*m. The fluid has a constant density (*ρ*) and dynamic viscosity (*μ*). The air inside the bubble has negligible density and viscosity. The momentum and continuity equations for incompressible Newtonian fluid flow in dimensional forms are

$$\rho \left(\frac{\partial {\text{u}}^{*}}{\partial {\text{t}}^{*}}+{\text{u}}^{*}\cdot \nabla^{*}{\text{u}}^{*}\right)=\nabla^{*}\cdot \left(-{\text{p}}^{*}\text{I}+\mu \left(\nabla^{*}{\text{u}}^{*}+\nabla^{*}{{\text{u}}^{*}}^{\text{T}}\right)\right)+\rho g,$$ (1)

$$0=\nabla^{*}\cdot {\text{u}}^{*},$$ (2)

where I is the unit identity matrix, u^*^ is the fluid velocity, p^*^ is the pressure, t^*^ is the time, *ρ* is the density, *μ* is the dynamic viscosity, *g* is the acceleration of gravity, and T is the transpose of the matrix.

Using R, U, *μ*U/R, and R/U to scale length, velocity, pressure, and, time, respectively, Eqs. (1) and (2) can be rewritten in the dimensionless form as

$$\text{Re}\left(\frac{\partial \text{u}}{\partial \text{t}}+\text{u}\cdot \nabla \text{u}\right)=\nabla \cdot \sigma +\frac{\text{Bo}}{\text{Ca}},$$ (3)

0=∇⋅u, (4)

where the dimensionless numbers Re, Bo, and Ca are given by

$$\text{Re}=\frac{\rho U\text{R}}{\mu },\text{ Bo}=\frac{\rho g{\text{R}}^{2}}{\gamma },\text{ Ca}=\frac{\mu U}{\gamma },$$

and the Cauchy stress tensor σ is

$$\sigma =-\text{pI}+\left(\nabla \text{u}+{\left(\nabla \text{u}\right)}^{\text{T}}\right).$$ (5)

Throughout this study, the slow viscous fluid flow (Re ≪ 1) was considered in the microvasculature, for which the inertial term in Eq. (3) maybe neglected. The final system for incompressible Newtonian fluid flow is

$$0=\nabla \cdot \sigma +\frac{\text{Bo}}{\text{Ca}},$$ (6)

0=∇⋅u (7)

in a fluid domain ∏ with a boundary Γ.

### A. Natural and essential boundary conditions

A parabolic velocity profile of mean velocity *U* was specified at the inlet and outlet of the channel to ensure the mass balance, while a no-slip boundary condition at the walls. The domain inside the bubble was not simulated in this work,^15,25^ and so no boundary condition exists. At the free surface, the following dimensionless normal stress boundary condition holds:^15,26^

$$\sigma \cdot \text{n}=-{\text{P}}_{\text{a}}\text{n}+\frac{1}{\text{Ca}}\left(\nabla_{\text{s}}\cdot \text{n}\right)\text{n,}$$ (8)

where ∇_*s*_ is the surface gradient operator defined as ∇_*s*_ = (I − nn) · ∇, n is the unit normal vector from fluid to air, and P_a_ is the air reference pressure. Equation (8) is a natural boundary condition to be applied during variational formulation. The last one is the kinematic boundary condition given by

u⋅n=0. (9)

In COMSOL, Eq. (9) is directly applied as a “weak constraint” on the free surface.

### B. Variational formulation

Equations (6) and (7) were numerically solved using FET in COMSOL. To ensure that the mixed weak formulation of Eqs. (6) and (7) is well-posed and stable, the Ladyzhenskaya–Babuska–Brezzi (LBB) or inf-sup condition should be satisfied.^27–29^ To fulfill this condition, quadratic and linear piecewise polynomials or shape functions were selected for velocity and pressure, respectively,^30^ defining quadratic and linear shape functions as Φ and ψ, respectively. To obtain the weak or variational forms, multiplying Φ and ψ with Eqs. (6) and (7), respectively, and integrating over the spatial domain ∏ must be carried out. This gives

$$0=\int_{\prod }\Phi \left(\nabla \cdot \sigma +\frac{\text{Bo}}{\text{Ca}}\right)d\prod ,$$ (10)

$$0=\int_{\prod }\psi \nabla \cdot \text{u}d\prod .$$ (11)

Equations (10) and (11) are the weighted-integral forms of Eqs. (6) and (7), respectively. Integrating by parts Eq. (10) and re-arranging give

$$\int_{\prod }\nabla \Phi \cdot \sigma d\prod -\frac{\text{Bo}}{\text{Ca}}\int_{\prod }\Phi d\prod =\int_{\prod }\Phi \left(\sigma \cdot \text{n}\right)d\prod .$$ (12)

Using Eq. (8) and application of the surface divergence theorem^26^ results in

$$\int_{\prod }\nabla \Phi \cdot \sigma d\prod -\frac{\text{Bo}}{\text{Ca}}\int_{\prod }\Phi d\prod =\frac{1}{\text{Ca}}\int_{\Gamma }\nabla_{s}\Phi d\Gamma ,$$ (13)

where the normal stress boundary condition, given in Eq. (8), has been used on the free surface boundary Γ. In the derivation of Eq. (13), the contour integral has been neglected because the bubble propagation has a zero contact line. In addition, the air reference pressure was assumed to be zero. In this analysis, a negligible term Re*∂*u/*∂*t in Eq. (13) at the left-hand side was also maintained for time marching, which vanishes at the steady state. Many methods have been previously used to track the interface of the propagating bubble.^31–35^ However, these methods are highly computationally expensive and time-wasting. COMSOL has significantly reduced these efforts. In the current study, the right-hand side of Eq. (13) is applied as a “weak contribution” in COMSOL to directly track the interface of the propagating bubble at each time step. Finally, Eqs. (11) and (13) are solved with the help of Arbitrary Eulerian–Lagrangian (ALE) moving a mesh application mode on a freely moving deformed mesh, which constitutes the fluid domain.

### C. Definition of parameters

The model contains two important parameters, i.e., Ca and Bo. It is necessary to specify the values of these parameters in the range that can seriously damage the pulmonary epithelium between the terminal and respiratory bronchioles during ventilation as the cells in this region are more susceptible to injury. The expected airway radius R and total cross-sectional area^23,36^ found in the terminal and respiratory bronchioles range from 0.15 mm to 0.5 mm and 100 cm^2^ to 1000 cm^2^, respectively. For normal breathing conditions of tidal volume equal to 500 ml and 12 breaths/min, typical small reopening velocities are studied in the literature range from 1 mm/s to 10 mm/s.^13^ Assuming the fluid viscosity and air–liquid surface tension as 47 cP and 72 dyn/cm, respectively, the corresponding Re, Ca, and Bo are then given by

$$3.2\times 10^{-3}\leq \text{Re}\leq 0.1,\;6.5\times 10^{-4}\leq \text{Ca}\leq 6.5\times 10^{-3},$$

and

$$3.1\times 10^{-3}\leq \text{Bo}\leq 3.4\times 10^{-2}.$$

However, for lung fluids, having surface tension much lower than 72 dyn/cm^12^ would lead to larger Ca and Bo values.^15^ To investigate all these possible Ca and Bo values that may exist in the human lungs, inertial effects were neglected, and about baseline conditions (i.e., Ca = 0.06 and Bo = 0.003), parameter independent variation studies were then performed for a range of Ca and Bo values.

### D. Convergence and re-meshing strategy

To achieve equilibrium (steady-state solution), a smoothed step function feature was created to ramp up the inflow velocity. A time step size of Δt = 0.001 s, while relative and absolute tolerances of 0.0001 and 0.000 01, respectively, with a maximum number of 50 iterations were also specified. The computational domain was fine-meshed with 10 996 quadratic triangular elements. A dense mesh is used near the gas–fluid interface to achieve correct results during bubble propagation. To avoid the inverted/twisted mesh elements during the calculation, a re-meshing strategy with a Winslow smoothing method was also adopted. To make it more understandable, for a moving mesh with the quality of an element below than 0.1, were considered as a worst, automatic re-meshing procedures were applied to re-mesh the geometry with a good quality of elements and computations were continued.

### E. Mesh independence analysis

A mesh independence study was also conducted to evaluate the correct mesh size for mass conservation and hydrodynamic stresses. According to the law of conservation of mass, the flow rate in the parent channel must be equal to the sum of the flow rates in the daughter channels if there is no change in bubble volume. Mathematically, *Q* = *Q*_1_ + *Q*_2_, where *Q* is the total flow rate in the parent channel and *Q*_1_ and *Q*_2_ are the flow rates in the lower and upper daughter channels, respectively. To satisfy this condition, two different mesh sizes were examined. For each mesh size, the inlet and outlet flow rates and bubble volume change were compared. By checking mesh statistics, initially, 10 996 quadratic triangular elements were used. Then, an extra-fine mesh of 16 466 elements was simulated, and the mass conservation difference and bubble volume change were recorded to be less than 1% and 0.5%, respectively. For both mesh densities, the difference in pressure and shear stress gradient magnitudes was found to be less than 1%.

## III. RESULTS

### A. Effect of Bond number

**FIG. 2.**
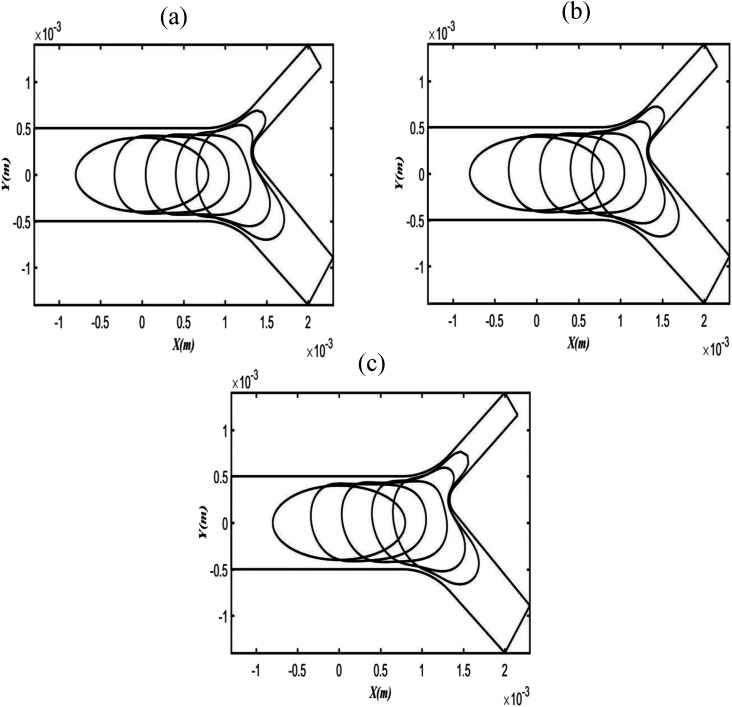
Microbubble profile during propagation in the asymmetric bifurcating airway at different Bo values with fixed Ca = 0.06 and time, t = 0.037 s. Bo = 0.003 (a), Bo = 0.25 (b), and Bo = 0.5 (c).

In this study, the influence of gravity on pressure gradients, shear stresses, and shear stress gradients is analyzed by varying Bo from 0.003 to 0.5 with fixed Ca = 0.06. The airway is asymmetrically bifurcated such that the diameter of the lower daughter airway is two times that of the upper daughter airway. Figure 2(a) shows that initially, at a low Bo value (i.e., Bo = 0.003), a large volume of bubbles penetrates the lower daughter airway (larger diameter airway) and a thin bubble nose moves to the upper daughter airway (smaller diameter airway). However, as Bo increases, the buoyancy force pushes the bubble to the upper narrow daughter airway, and consequently, there is an increase in the volume of the thin bubble nose, as shown in Figs. 2(b) and 2(c).

**FIG. 3.**
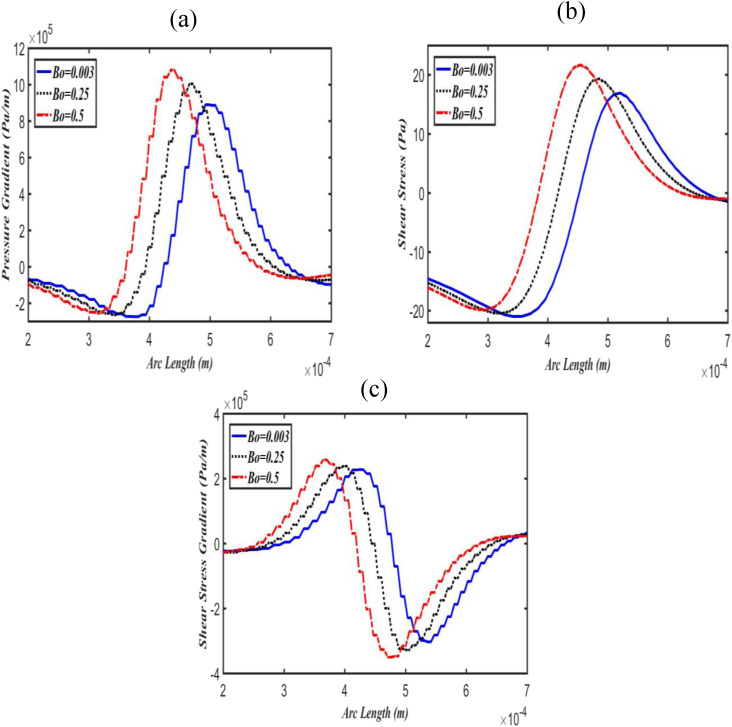
Effect of Bo on pressure gradient (a), shear stress (b), and shear stress gradient (c) along with the upper daughter airway wall at fixed Ca = 0.06.

**FIG. 4.**
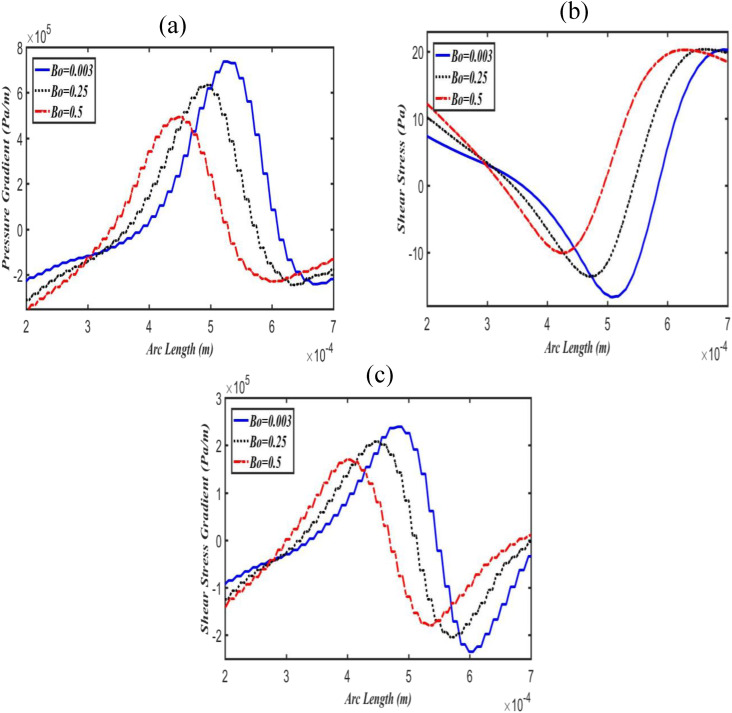
Effect of Bo on pressure gradient (a), shear stress (b), and shear stress gradient (c) along with the lower daughter airway wall at fixed Ca = 0.06.

The effects of Bo on pressure gradients, shear stresses, and shear stress gradients along the upper and lower daughter airway walls are shown in Figs. 3 and 4, respectively. Figure 3(a) shows that the pressure gradient is initially (i.e., at zero arc length) very low; however, as it penetrates to the upper daughter airway, there is a peak in the pressure gradient at a point approximately (0.0005, 0). It also reveals that as Bo increases, the peak in the pressure gradient also increases in the upper daughter airway wall, while it decreases in the lower airway wall, as can be seen in Fig. 4(a). Similar patterns were also observed for shear stress and its gradients [Figs. 3(b), 3(c), 4(b), and 4(c)] in the upper and lower daughter airway walls. However, these results indicate that the magnitudes of the shear stress and its gradients are significantly low compared to the pressure gradient. This strongly suggests that the pressure gradient is a potential component of hydrodynamic stresses, which can seriously damage the pulmonary epithelium.

### B. Effect of capillary number

**FIG. 5.**
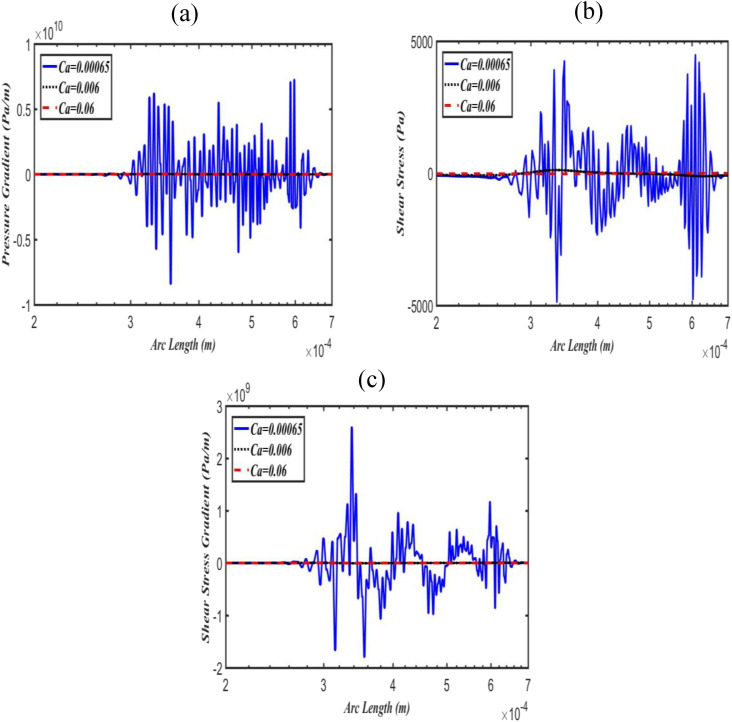
Effect of Ca on pressure gradient (a), shear stress (b), and shear stress gradient (c) along with the upper daughter airway wall at fixed Bo = 0.003.

**FIG. 6.**
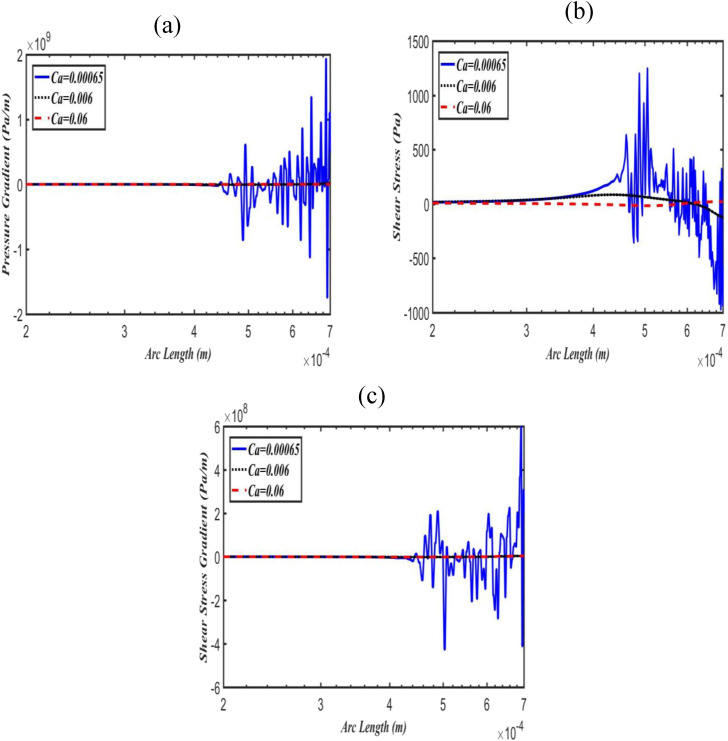
Effect of Ca on pressure gradient (a), shear stress (b), and shear stress gradient (c) along with the lower daughter airway wall at fixed Bo = 0.003.

To investigate the effects of surface tension on pressure gradients and shear stress and its gradients, Ca was computed for different values (i.e., 0.000 65, 0.006, 0.06) with constant Bo = 0.003. It is observed that at low Ca, the bubble is almost circular, leaves a thin liquid layer with the parent airway wall, and splits negligibly to the leading daughter branches. For larger Ca, the bubble is elongated and adds more fluid to the thin liquid layer. Results indicate that at larger Ca, the bubble penetrates significantly to both the daughter branches with thicker film thickness. Moreover, Ca has a remarkable effect on pressure gradients, shear stresses, and shear stress gradients compared to gravity. Figure 5(a) shows that even at low Bo, for Ca = 0.000 65, there is a large and complex cycle of pressure gradient near the thin bubble tip in the upper daughter airway wall, which rapidly decreases as Ca increases. Interestingly, increasing Ca leads to a decrease in pressure gradients in both the leading daughter walls. In addition, the similar fashions of shear stress and its gradients were noted for Ca in both the upper and lower daughter airway walls [Figs. 5(b), 5(c), 6(b), and 6(c)]. More importantly, the pressure gradient has a very large magnitude in comparison to the shear stress gradient.

## IV. DISCUSSION

It is now clear that mechanically ventilating patients suffering from ARDS can complicate existing cell damage. In this analysis, a finite element numerical approach was used to investigate the effects of Bo and Ca on pressure gradients, shear stresses, and shear stress gradients in a 2D asymmetric bifurcating airway model. In previous experimental studies,^37–39^ Bo was simulated in the range from 0 to 1.26. In the case of computational work,^15^ Bo is estimated (between the conducting and respiratory bronchioles) by assuming the airway as symmetrically bifurcating in the range from 0.068 to 1.36, and for larger Bo (i.e., Bo > 1), the bubble tends to split unequally. For the problem in hand, Bo can be approximated from 0.0031 to 0.5 as lung fluids may have different surface tensions (see Sec. II C). In the current analysis, the effective value of Bo is 0.25 at which the bubble starts to elevate the upper narrow daughter branch and is much less than 1. It shows that the range of Bo varies in the airway tree, and it is difficult to estimate the range of Bo for present work from the previous study where symmetrically bifurcating airway was analyzed.^15^

In present numerical simulations, Bo was varied from 0.0031 to 0.5 with fixed Ca = 0.06. Interestingly, at low Bo, the bubble penetrates to the lower gravity favor branch, while it elevates dramatically to the upper daughter branch for Bo ≥ 0.25 due to buoyancy forces (Fig. 2). It is also found that increasing Bo results in an increase in pressure gradients in the upper daughter airway wall, while a decrease in the lower, as shown in Figs. 3(a) and 4(a). These results are consistent with the previous results,^15^ where bubble propagation was simulated in symmetrically bifurcating airways. Chen *et al.* proved that at low Bo, the bubble always splits symmetrically, and the magnitudes of pressure and shear stress gradients are identical. However, the asymmetric bubble flows are unique from symmetric bubble propagation due to two major reasons found here. First, the microbubble volume that penetrates to the daughter branches is always unequal even if the buoyancy force is dominant (Fig. 2). Second, the magnitudes of hydrodynamic stresses including pressure and shear stress gradients are also always larger in the upper daughter airway wall compared to lower daughter airway wall even at low Bo (see Figs. 3 and 4). The reason for larger hydrodynamic stresses in the upper daughter airway is that the magnitudes of hydrodynamic stresses are inversely proportional to airway radius.^13,22,25^ Results demonstrate that the pressure gradient magnitudes are much larger than the magnitudes of shear stress gradient. The pressure gradient is the potential component of hydrodynamic stresses to damage pulmonary epithelium. It also shows that for buoyancy-driven flows in bifurcating airways, the lower daughter airway wall is safer from mechanical stresses related injuries than the upper daughter airway walls.

Previously, many researchers used computational techniques to simulate microbubble propagation in straight and bifurcating airways for a wide range of Ca values (i.e., 0.0008 ≤ Ca ≤ 1).^15,25,40^ In addition, in *in vitro* experimental conditions of airway reopening, Ca varies in the range 0.0001 ≤ Ca ≤ 0.01.^10,11,18^ In this work, as in Sec. II C, Ca was simulated in the range 0.000 65 ≤ Ca ≤ 0.06, which may exist in the human’s deep lungs (i.e., between the terminal and respiratory bronchioles). The effect of Ca on pressure gradients, shear stresses, and shear stress gradients in the bifurcating daughter airway walls has been shown in Figs. 5 and 6. Figure 5(a) shows that at low Ca, there is a large and complex cycle of pressure gradient near the thin bubble cape in the upper daughter airway wall. As Ca increases, the magnitude of the pressure gradient dramatically decreases. Figure 6(a) indicates that as Ca increases, the pressure gradient has also the same patterns in the lower daughter airway wall as well. The results presented here strongly agree with the previously published data by many authors.^10,13,15^ Again, the magnitudes of pressure and shear stress gradients are much larger in the upper daughter airway wall, which supports the result that the magnitudes of hydrodynamic stresses are inversely proportional to the diameter of the airway. The magnitude of the pressure gradient in the upper daughter airway wall is more sensitive to Ca compared to Bo. These results suggest that the cell damage has a strong correlation with a pressure gradient, weak with shear stress gradient and negligible with shear stress.^23^ On the basis of these results, one can easily conclude that the upper daughter airway wall would experience more cell necrosis compared to the lower.

Besides, as this computational model presented significant effects of gravity and surface tension on the magnitudes of the pressure gradient in the bifurcating airways, this model also has two theoretical predictions that could be examined in future experimental studies on the asymmetric bifurcating airways. First, as shown in Fig. 2, a small volume of the bubble moves to the upper daughter branch as Bo increases, and the magnitudes of pressure gradient are much larger in the upper daughter airway wall than the lower even at low Bo, and this implies that the upper daughter airway wall would experience more cellular injuries. Second, for each low or large dimensionless bubble velocity Ca, the upper narrow daughter airway wall is more prone to cell necrosis.

## V. CONCLUSIONS

In this paper, an asymmetric bifurcating airway model was proposed, where the diameter of the upper daughter airway is half of the lower. Finite element procedures were utilized and solved Stokes’s equation to simulate steady microbubble propagation in the asymmetric bifurcating airways for different Bo and Ca values. This computational model has some simplifications that limit its direct applicability and its comparison with the real *in vivo* system. In this model, the mucus fluid is Newtonian with constant density and viscosity. However, in the human pulmonary system, the mucus fluid is non-Newtonian and has different types of properties such as shear-thinning, viscoelasticity, and more specifically non-zero yield stress (i.e., 400–600 dyn/cm^2^ for healthy lungs and higher for diseased), which results in hydrodynamic stress amplification and cell death.^41–44^ The surface tension is assumed to be constant, while in the pulmonary system at the gas–liquid interface, spatial and temporal gradients of surface tension exist in the presence of pulmonary surfactants.^20,45,46^ In this analysis, the role of surfactants has also been neglected; however, earlier studies have shown that surfactants reduce the hydrodynamic stresses and play a key role in cell protection.^20,47,48^ Although the airway’s walls are rigid, in the pulmonary system, the airway’s walls are elastic/compliant, and during the reopening of collapsed pulmonary airways, large magnitudes of inward-directed pressure gradients exist, which can further damage epithelial cells.^49–51^ Finally, this model does not include any live cells cultured on the airway wall, while there are uncountable live cells in the pulmonary system.^10^

Although this computational model has several limitations, this idealized asymmetric bifurcating airway model has provided much important new information about microbubble propagation/splitting and epithelial cell damage. In the future, additional computational studies on bifurcating airways are required to better understand the pulmonary mechanical response.

In conclusion, this computational study aimed at elucidating the effects of gravity and surface tension on microbubble propagation and pressure and shear stress gradients. Results indicate that although a small fraction of the bubble penetrates the upper daughter airway as Bo increases, the magnitudes of the pressure gradient are much larger than of the lower. Similarly, in the case of low bubble velocities (i.e., low Ca), there is a large and complex cycle of the pressure gradient in the upper daughter airway wall. These results imply that in a lung’s deep asymmetric bifurcating airways, the daughter airway with a smaller diameter is more susceptible to injury compared to the larger diameter daughter airway as the magnitudes of hydrodynamic stresses are inversely proportional to the airway diameter. This study indicates that Ca has a greater impact on the magnitudes of the pressure gradient than Bo. This computational analysis has, therefore, listed important novel information about the understanding of the mechanism of epithelial cell injury in complex bifurcating geometries.

## DATA AVAILABILITY

The data that support the findings of this study are available from the corresponding author upon reasonable request.

## Figures and Tables

**FIG. 7.**
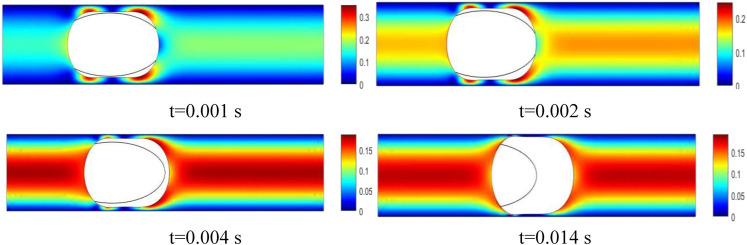
Bubble position and velocity profile during propagation in a fluid-filled straight channel at different times. The black solid line indicates the initial position of the bubble.

## APPENDIX: VALIDATION OF THE NUMERICAL METHOD

The numerical procedures were first validated by simulating microbubble propagation in a straight 2D fluid-filled channel. To do this, the unsteady incompressible Navier–Stokes equations with negligible volume force were solved with the help of the ALE moving the mesh application mode in COMSOL. The parabolic velocity profile with a mean velocity of *U* was specified at the upstream and downstream, while no-slip boundary conditions at the walls of the channel. Using direct interface tracking [right-hand side of Eq. (13)] and re-meshing techniques, initially from the perfectly elliptical shape, the bubble attains a steady-state position, as shown in Fig. 7. This problem was first studied by Bretherton^52^ for low Ca values. The code was also verified by reproducing the results of Chen *et al.*^15^ shown in Fig. 7.
